# Combining Structure and Sequence Information Allows Automated Prediction of Substrate Specificities within Enzyme Families

**DOI:** 10.1371/journal.pcbi.1000636

**Published:** 2010-01-08

**Authors:** Marc Röttig, Christian Rausch, Oliver Kohlbacher

**Affiliations:** 1 Center for Bioinformatics Tübingen, Eberhard-Karls-Universität Tübingen, Tübingen, Germany; 2 Chair of Biological Chemistry, Technische Universität München, Freising-Weihenstephan, Germany; University of Oxford, United Kingdom

## Abstract

An important aspect of the functional annotation of enzymes is not only the type of reaction catalysed by an enzyme, but also the substrate specificity, which can vary widely within the same family. In many cases, prediction of family membership and even substrate specificity is possible from enzyme sequence alone, using a nearest neighbour classification rule. However, the combination of structural information and sequence information can improve the interpretability and accuracy of predictive models. The method presented here, Active Site Classification (ASC), automatically extracts the residues lining the active site from one representative three-dimensional structure and the corresponding residues from sequences of other members of the family. From a set of representatives with known substrate specificity, a Support Vector Machine (SVM) can then learn a model of substrate specificity. Applied to a sequence of unknown specificity, the SVM can then predict the most likely substrate. The models can also be analysed to reveal the underlying structural reasons determining substrate specificities and thus yield valuable insights into mechanisms of enzyme specificity. We illustrate the high prediction accuracy achieved on two benchmark data sets and the structural insights gained from ASC by a detailed analysis of the family of decarboxylating dehydrogenases. The ASC web service is available at http://asc.informatik.uni-tuebingen.de/.

## Author Summary

Prediction of enzymatic function of experimentally uncharacterised sequences is an important task in annotation of sequence databases. While all the information on an enzyme's specificity is necessarily contained in its sequence, prediction methods using sequence alone often do not perform all that well. Obviously, structural information – if available – will yield precious hints on the function and relative importance of specific sequence positions with respect to substrate specificity. We propose a novel method (Active Site Classification, ASC) for enzyme classification bringing together structural information and sequence information. Our ASC web server allows users to build predictive models in an automated way focused on relevant enzyme residues and furthermore to interpret the models to gain insights into the mechanism of enzyme substrate specificity.

## Introduction

The number of protein sequences in public databases has been growing almost exponentially due to great advances in sequencing technologies and a decline in sequencing costs. However, as the number of experimentally characterised sequences does not grow at the same speed, the fraction of protein sequences without any functional annotation also increases. An experimental investigation of all these new sequences would be too time-consuming and too costly. Consequently, the fraction of enzyme sequences in current databases with experimentally characterised function is around 5% only [1]. Therefore, automated computational methods are needed to assign a putative function to uncharacterised sequences reliably.

To predict the putative function of a protein, currently established methods rely on the fact that two proteins with similarities between their sequences have similar structures and also a similar function [2]. An uncharacterised enzyme sequence can often be associated with a putative function by searching against sequences of functionally characterised enzymes, using BLAST [3] or Hidden Markov Models (HMMs) [4],[5] and annotating the query sequence with the function of the best hit.

There were several studies that tried to find a critical point for the minimum degree of similarity that is required to make a transfer of function safe when using such sequence-based tools to infer enzymatic function by similarity [6]–[8]. In general, one can assume an accuracy of at least 90% when transferring the function, as defined by the full enzyme commission (EC) number, between sequence pairs that have at least 60% sequence identity [1],[8]. For sequence identities below 60% the accuracy decreases quite rapidly, making transfer of function by homology increasingly error prone. At 40% sequence identity the accuracy of function transfer already dropped to 50%. This is due to the fact that at high sequence identity, most pairs are genuine orthologous sequence pairs. However, at a lower sequence identity the probability that paralogs get paired with the query sequence increases, because the nearest ortholog to the query sequence might be missing in the set of annotated sequences and so the nearest paralog is chosen instead. This was also pointed out by Chen and Jeong [9], who concluded that the crucial task of finding annotated orthologous sequences to infer enzyme function should be solved using detailed information about the residues lining the active site or other residues identified by experiment. They argued that in this way one can reliably separate orthologs from paralogs, thereby making the transfer of functional annotation safe.

Incorporation of structural information into the process of building a predictive model for enzyme specificity was also suggested by Stachelhaus *et al.* [10] and Challis *et al.* [11] for Nonribosomal Peptide Synthetases (NRPS). They tried to infer a specificity-conferring code of the active site, which could predict what substrate would be used, based on the residues lining the active site and sequence data. Subsequently, Rausch *et al.* combined these approaches with Support Vector machines (SVMs) for classification and achieved significantly improved prediction performance [12].

Similarly, the EFICAz method devised by Tian *et al.* [13] operates on functionally discriminating residues (FDR) that are detected by analysing homofunctional and heterofunctional multiple sequence alignments (MSAs) of members of an enzyme family using information theoretic measures. However, no structural information is used when determining those FDRs. Prediction of enzyme function is then based on these FDRs and combined with PROSITE pattern matches and familiy specific thresholds for sequence identity.

The recent extension of this method named EFICAz2 further added a SVM-based component built upon the positions of the query sequence aligned to the MSA of the enzyme family [14] and also a final Decision Tree model to increase the prediction accuracy when making predictions for sequences that are more distant to the training set. An approach similar to the FDR-based method of EFICAz was devised by Hannenhalli and Russell and based predictions of class membership only on those columns of a family MSA that had a positive relative entropy (sub-profile method) [15].

In this work, we propose a generalisation of the approach by Stachelhaus and Challis to base function predictions on active site residues and make it amenable to any enzyme family that has associated structural information. Thus, classification errors induced by using only the simple descriptor of global sequence identity, which often fails when predicting more distant sequences, could be ameliorated by the incorporation of additional structural information about the active site configuration of the enzymes. Our new approach, called Active Site Classification (ASC), can be used for a family of enzyme sequences where all members perform the same type of reaction but with different substrates. The only requirement is a training set of annotated sequences and one homologous crystal structure. The program will then train a SVM model that can be used to predict the specificity of unclassified sequences. Furthermore, the model can be used to infer which residues and properties are important for each sub-specificity.

In the following sections we will show the very good performance of ASC on two benchmark data sets and that the performance gain is achieved by concentrating on the active site residues. We will also show how the determined ASC model can be used to learn more about the importance of each active site residue and interpret their putative function in the structural context of the active site.

## Materials and Methods

### Acquisition of sequence data

All sequences used in this study were extracted from the UniProt release 15.8 from September 2009 [16]. SwissProt served us as a source of reliable EC number assignment for the training sequences used in this study. Sequences annotated as fragment, tagged as probable or annotated with multiple EC numbers were removed.

### Benchmark data sets

The first benchmark data set was used by Hannenhalli and Russel [15] to evaluate their sub-profile method and is a set of enzyme classification problems that have clearly defined subtypes, which are not easily discernible by sequence comparison or phylogenetic analysis of the MSAs. This data set contains nucleotidyl cyclases (EC 4.6.1.1, 4.6.1.2), eukaryotic protein kinases (EC 2.7.11.-, 2.7.10.-), trypsin-like serine proteases (EC 3.4.21.70, 3.4.21.71, 3.4.21.1, 3.4.21.4) and lactate/malate dehydrogenases (EC 1.1.1.27, 1.1.1.37). For the kinase family we used the alignments of the Protein Kinase Resource [17] and all other sequences were retrieved from SwissProt according to their EC number.

The second benchmark was extracted from a data set compiled by Capra and Singh [18]. Their data set contains MSAs of EC annotated sequences that share a specific Pfam domain. Each MSA contains two subtypes of an enzyme family, which share the first three digits but differ in the fourth digit. Furthermore, columns within the MSAs are also annotated whether they are in proximity to a substrate in a representative template structure, the distance cutoff is 5 Å. With this information we could extract the active site residues and train our SVM models to benchmark ASC on this data set. Of the original 284 pairs of subtype MSAs we selected all MSAs with at least 15 sequences in each class to be able to train statistical meaningful models during model validation. After this filtering step we had 48 MSAs for benchmarking ASC.

### 1-NN classifier

The nearest neighbour classifier (1NN) is an instance-based classifier. The 1NN classifier makes predictions for a test data point 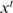 by searching for the nearest data point 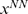, according to some distance function, in the training set and reports the label of 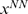 as the predicted label for the test data point 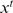. The distance between two enzymes is defined as the number of mismatching residues in a sequence alignment of the complete enzyme sequences.

### Support vector machines

SVMs are supervised classification models that can be used to train a classification model on a given set of 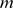 training samples 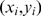 where 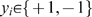 is the class label of the data point 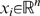. The SVM model consists of a hyperplane that partitions 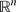 into two half-planes, one for each class. The functional form of the SVM model is given by 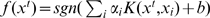 in its dual formulation. The hyperplane is determined using the maximum-margin concept, thereby making the SVM model per design more resilient to overfitting than many other classification models [19]. The training problem of the SVM model in its dual formulation can be solved efficiently using quadratic programming routines or specialised SVM libraries. We used the popular LIBSVM implementation [20] to train the SVM models. Another advantage of SVMs is the easy step from linear to non-linear classification models by the use of kernels [21]. Multiclass classification problems are solved by training 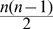 pairwise classification models 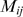 and then using a test sequence with all pairwise models 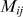 that give votes for either class 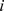 or class 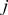. In a final step, all votes are summed and the class receiving most of the votes is the predicted class label.

### Sequence kernels

Kernel methods like SVMs can also operate directly on the objects to classify without prior encoding of these objects into numerical feature vectors. This is made possible by the use of kernel functions that are defined on those objects and represent a similiarity measure. Within ASC we make use of several kernel functions that are defined on the set of signature sequences. The first one is a simple string kernel 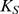 that uses the number of matching symbols as a similarity measure. The second kernel is the BLOSUM kernel 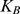 that uses the summed similarity score of the BLOSUM62 matrix between pairs of signature sequences as a kernel. The BLOSUM62 matrix 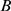 has to be transformed from its log-odds form back into the substitution probability form 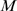 [22] to construct a valid Mercer kernel 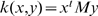 defined on aligned pairs of amino acids, where 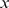 and 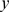 are indicator vectors encoding the two aligned amino acids. The kernel 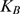 on sequences can then be defined by summing the kernel values 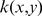 over all aligned positions of two signature sequences [23]. The third kernel 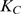 can be defined by using the positive semi-definite chemical similiarity matrix MCLA720101 from the AAindex database instead of the transformed BLOSUM matrix [24]. Our last kernel 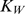 is defined by encoding each signature amino acid into three descriptors 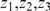 and using the scalar product between the feature vectors in the space of encoded sequences as a kernel function. The descriptors were derived by Wold *et al.* [25] and encode information about hydrophobicity, size and electronic properties. For comparison purposes we also made use of the RBF kernel defined on the full MSA as introduced by Arakaki *et al.* [14].

### Interpretation of SVM models

Importance of specific signature positions for the classifcation models can be either quantified by calculating the primal weight vector 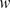 of the SVM model and then sort the weights by absolute magnitude to get a ranking of the importance of each variable and its corresponding signature position. This can only be done with kernels having a primal representation, like the Wold kernel 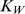 and the simple string kernel 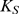. For kernels that are defined in the dual space only, one can quantify the influence of a signature position by restricting kernel computation to a single column of the signatures during a full cross-validation (CV) run. The importance of each position is then given by its achieved classification accuracy. In a multiclass setting the accuracy of each pairwise classification model 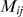 is taken as the score of a signature position for discriminating between the two respective classes.

### Training and performance measures

We evaluated the performance of our ASC method in a fashion similar to the evaluation of the EFICAz method by ensuring that the training set sequences are not too similar to the test sequences (according to sequence identity) [14]. The similarity of the test and training set sequences is controlled by the maximum training to testing sequence identity (MTTSI). We used the sequence identity computed over all aligned residues as similarity measure. The models were trained using nested cross-validation (CV). The outer CV-loop leaves one data point out and does model-selection on all remaining data points that do not have a sequence identity higher than a given MTTSI value by using 5-fold cross-validation in the inner loop. The model-selection routine in the inner loop searches for the hyperparameter 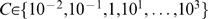 that yields the best SVM model using only the retained data. Model selection is guided by the accuracy estimated in the inner CV-loop. The selected model is then used to predict the class of the left-out data point. The generalisation capability of the SVM model is estimated by computing for each class the 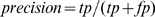 and 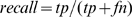 and averaging these statistics over all classes. The overall accuracy of a model is then given by the harmonic mean of these two measures 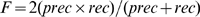, called the F-measure.

### The ASC method

The ASC method utilises SVMs to build a model that discriminates between two or more classes of enzymatic activity. It starts with training data in the form of sequences from each sub-specificity class and a PDB structure [26] that serves as a structural template. The location of the enzyme's active site is identified by specifying a certain residue or substrate contained in the template PDB structure. If there is no co-crystal structure with substrate available, but the spatial location of the active site is known, three dimensional coordinates can be supplied alternatively to define the active site.

**Figure 1:**
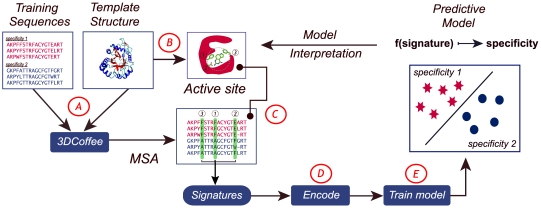
Graphical overview of the ASC method. (A) In the first step training sequences are aligned using 3DCoffee to get an MSA. (B) In a second step residues lining the active site are extracted from the template structure. (C) The third step maps the extracted residues along the MSA to get a signature of the active site for each sequence. (D) These signatures are then encoded into feature vectors using the three descriptors 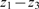. Alternatively, kernels may be used. (E) The final ASC model is trained using the generated feature vectors.

In a preprocessing step, all sequences with a sequence identity to the template that is too low (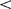
20%) are discarded. In the first step the five most diverse sequences from each class are chosen and aligned using 3D-Coffee [27] to get a guide MSA to which all other sequences are subsequently aligned (Figure 1A). In the second step, all residues that are close enough to the active site of template structure (normally within 6 Å) and receive a high CORE score by 3D-Coffee (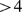) are extracted (Figure 1B) [28]. These signature subsequences from each sequence represent residues occupying spatially equivalent regions in the active site (Figure 1C). The active site signatures are then transformed into numeric feature vectors by encoding each amino acid from a signature into the three descriptors 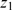, 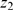 and 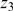 (Figure 1D). Gaps are encoded by three zeros. The resulting labelled feature vectors are then used to train a SVM to get the final classification model (Figure 1E). Alternatively, kernel functions can be used to work directly on the signature sequences. The weights are sorted according to their absolute magnitudes, yielding a ranking of the importance of the active site residues. Residues whose descriptors receive higher weights are more important for the classification of the enzymes.

## Results/Discussion

Previous studies already showed that functional annotation of a test sequence is easy, if the sequence identity to an annotated sequence is above 60%, since the probability of error when transferring the annotated function of the hit to the query sequence is rather low [8],[14]. Thus, we assessed the performance of ASC by trying to predict the function of test sequences that are more distant to the training set. Therefore, we choose a MTTSI that is generally below 60%. However, the exact value where function annotation becomes trivial depends also on the enzyme family under study. Tian *et al.* [8] already defined family-dependent sequence identity thresholds (SIT) for each EC class where annotation based on sequence identity becomes very reliable. Thus, we tried to choose the MTTSI threshold below these determined SITs. However, we also had to ensure that the MTTSI is not too low to have enough training sequences to build our ASC models during the nested validation runs. We compared ASC to the 1NN classifier, serving as our baseline. The 1NN classifier can be seen as a more sophisticated BLAST classifier, since the alignment of the query is based on the full MSA and is not a simple pairwise alignment. To rule out that improvements of ASC over the 1NN classifier are not simply due to the use of SVMs, we also give the performance of the SVM classifier based on the whole MSA. This classifier can be seen as an ASC model with infinite cutoff distance. Arakaki *et al.* [14] already observed a performance increase when using SVM classifiers based on the MSA within EFICAz2 compared to the FDR-based classifier used in the first version of EFICAz. We applied all defined kernels, including the RBF kernel of EFICAz2, when building the ASC and MSA-based SVM models, and report only the performance of the best model. In this way we can also compare the performance of ASC to the best performing classification component of the EFICAz2 system by including the performance of the SVM based on the full MSA. In both benchmarks the performance is given by the F-measure achieved by the classifiers. Detailed tables containing F-measure, precision, recall and best performing kernels can be found in the supplementary material (Text S1, Text S2).

### Hannenhalli benchmark

Overall, the ASC model had three wins against the 1NN classifier and two wins against the SVM classifier based on the full MSA (Table 1). Thus, ASC clearly outperformed the sequence-distance based 1NN classifier, delivering very good performance in all four cases. Moreover, the classification improvement was achieved by using the active-site signatures instead of the full MSA, because the MSA-based model is also outperformed by the ASC model in two cases. The residues found most relevant for the ASC model were those that were already identified by experiment and also detected by the sub-profile method. We will describe the ASC results on the four enzyme families in more detail now.

**Table 1:** ASC performance on Hannenhalli benchmark.

Family	1NN	ASC	K	WDL	ASC	MSA	K	WDL	MTTSI	N
Cyclase	0.66	**0.97**	B	W	**0.97**	0.38	B	W	0.2	137
Kinase	**1.00**	**1.00**	S	D	**1.00**	**1.00**	S	D	0.4	294
Dehydrogenase	0.95	**0.98**	B	W	**0.98**	**0.98**	B	D	0.4	376
Trypsin	0.81	**0.90**	W	W	**0.90**	0.80	W	W	0.5	78

F-measures of the nearest neighbour classifier, the ASC classifier and the best classifier based on the full MSA are given in the columns 1NN, ASC and MSA, respectively. The first part of the table compares ASC with the 1NN classifier and the column WDL gives the wins, draws and losses of the pairwise comparisons. The best performing kernels are given in the columns labelled K. Similarly, the second part of the table compares ASC with the SVM classifier based on the full MSA. The last columns give the used MTTSI value and the number of available sequences.

### Protein kinase family

We used the crystal structure of the ternary complex of a protein kinase (PDB-Id: 1ATP, [29]) to determine the active site residues that are within 6 Å of the substrate ATP. The kinase family was modelled well with a maximal F-measure of 100% by all three methods. The 1NN classifier already achieved optimal performance on this family (Table 1). However, the ASC model also achieved optimal performance using only a fraction of the residues. The three most important residues for the ASC model were positions Thr201 (accuracy = 99.6%), Lys168 (accuracy = 99.2%) and Glu170 (accuracy = 97.7%). The importance of each signature position is given by the accuracy of the ASC model trained on solely this position. These residues were also detected by the sub-profile method devised by Hannenhalli and are known as putative modulating positions [30],[31].

### Nucleotidyl cyclase family

Clear performance improvements achieved by the ASC method over the two baseline classifiers can be seen for the cyclase family. The ASC model was built using the crystal structure of an adenyly cyclase (PDB-Id: 1AB8, [32]) and a distance cutoff of 5 Å. The 1NN classifier and the classifier based on the full MSA failed to achieve satisfying F-measures, whereas the ASC classifier achieved a very high F-measure of 97% (Table 1). The four most important residues, according to their influence on the ASC classification model, were Asp1018 (accuracy = 98.5%), Ile1019 (accuracy = 97.7%), Trp1020 (accuracy = 93.9%) and Lys938 (accuracy = 87.4%). These residues are known to have influence on the substrate specificity of nucleotidyl cyclases as shown by Tucker *et al.* [33]. These residues were also detected by the sub-profile method [15].

### Trypsin family

The ASC model for the trypsin family was built using a crystal structure of a serine protease (PDB-Id: 1AQ7, [34]) and a distance cutoff of 5 Å. The ASC classifier achieved a satisfying F-measure of 90%, whereas the 1NN and MSA-based classifier achieved a F-measure of roughly 80% only (Table 1). The three top-ranking residues that discriminate between chymotrypsin and trypsin were positions Asp189 (accuracy = 98.2%), Gly226 (accuracy = 92.4%), Gly219 (accuracy = 90%) and Ala221 (accuracy = 84.4%). Asp189, Gly226 and Ala221 were also detected by the sub-profile method and two of them, namely Asp189 and Ala221, are long known modulators of protease specificity [15].

### Malate/lactate dehydrogenase family

The ASC model for the dehydrogenase family was built using the crystal structure of a malate dehydrogenase from *E. coli* (PDB-Id: 1EMD, [35]) and a distance cutoff of 5 Å. Generally, this family was modelled well by all three classifiers with F-measures above 90% and there were only minor differences among the models. The residues with the greatest influence on the ASC model were Met227 (accuracy = 98.2%) and Arg81 (accuracy = 97.4%). Especially, the Arg81 residue is a proven specificity modulating position because mutating this residue to glutamine is known to confer lactate specificity [36].

### Capra benchmark

To get a better impression of the performance of our new method, we applied the ASC, 1NN and full MSA classifiers to a larger benchmark data set of 48 enzyme pairs extracted from the data set compiled by Capra and Singh [18]. Table 2 gives the performance of the classifiers as quantified by the F-measure. The first striking fact is that the 1NN classifier was quite competitive even in MTTSI ranges below 70%. There are 22 enzyme pairs where ASC and the 1NN classifier performed equally well. However, there were also 21 cases where the ASC model outperformed the 1NN classifier along with only 5 losses. The SVM models based on the full MSA were also quite competitive having 16 cases of equal performance with ASC and 8 cases where ASC performed worse. However, there are 24 cases where ASC outperformed the MSA-based classifier. The averaged F-measures of the MSA-based classifier, 1NN and ASC were 90%, 92% and 95%, respectively. Thus, the ASC method based on active site signatures is a clear step to more accurate function prediction especially for sequences with a greater distance to the training set. This is exemplified by the 11 cases where the ASC models could perfectly discriminate between the classes with a F-measure of 100% whereas the 1NN classifier clearly failed to achieve the same performance on these cases. Most often the simple string kernel 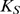 sufficed to build the best ASC or MSA-based model, with the other kernels showing similar performance. However, for some cases only one kernel excelled over the others. Some cases could be better modelled by the BLOSUM kernel, whereas other cases were better modelled by the Wold kernel 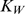. Thus, it is advisable to try all kernel functions when building ASC models and keep the best performing one.

**Table 2:** ASC performance on Capra benchmark.

EC pair	1NN	ASC	WDL	ASC	MSA	WDL	MTTSI
1.1.1.100/1.1.1.62	0.72	**0.76**	W	0.76	**0.85**	L	0.40
1.1.1.103/1.1.1.284	**1.00**	**1.00**	D	**1.00**	**1.00**	D	0.40
1.1.1.1/1.1.1.103	0.96	**1.00**	W	**1.00**	**1.00**	D	0.40
1.1.1.1/1.1.1.284	0.56	**0.82**	W	**0.82**	0.74	W	0.60
1.1.1.41/1.1.1.42	**0.94**	0.72	L	0.72	**0.94**	L	0.50
1.1.1.41/1.1.1.85	**1.00**	**1.00**	D	**1.00**	0.96	W	0.60
1.1.1.42/1.1.1.85	0.98	**1.00**	W	**1.00**	0.98	W	0.60
1.2.1.3/1.2.1.71	**1.00**	**1.00**	D	**1.00**	**1.00**	D	0.50
1.2.1.3/1.2.1.8	0.92	**0.93**	W	**0.93**	0.83	W	0.60
1.4.1.3/1.4.1.4	**1.00**	**1.00**	D	**1.00**	**1.00**	D	0.60
1.8.1.4/1.8.1.7	**1.00**	**1.00**	D	**1.00**	**1.00**	D	0.60
2.1.2.2/2.1.2.9	0.88	**1.00**	W	**1.00**	**1.00**	D	0.30
2.1.3.2/2.1.3.3	0.77	**1.00**	W	**1.00**	0.91	W	0.30
2.2.1.1/2.2.1.7	0.99	**1.00**	W	**1.00**	0.98	W	0.50
2.3.1.16/2.3.1.9	0.81	**0.93**	W	0.93	**0.96**	L	0.50
2.4.2.10/2.4.2.7	0.84	**1.00**	W	**1.00**	0.90	W	0.30
2.4.2.22/2.4.2.8	**1.00**	**1.00**	D	**1.00**	**1.00**	D	0.40
2.4.2.8/2.4.2.9	**1.00**	**1.00**	D	**1.00**	0.91	W	0.40
2.5.1.10/2.5.1.29	0.42	**0.75**	W	**0.75**	0.34	W	0.40
2.5.1.1/2.5.1.10	0.52	**0.58**	W	0.58	**0.65**	L	0.50
2.5.1.1/2.5.1.29	0.53	**0.77**	W	**0.77**	0.73	W	0.50
2.6.1.11/2.6.1.62	**1.00**	**1.00**	D	**1.00**	0.88	W	0.40
2.6.1.11/2.6.1.13	**0.94**	**0.94**	D	**0.94**	0.83	W	0.50
2.6.1.11/2.6.1.76	**1.00**	**1.00**	D	**1.00**	0.46	W	0.40
2.6.1.13/2.6.1.62	**1.00**	**1.00**	D	**1.00**	**1.00**	D	0.40
2.6.1.13/2.6.1.76	**1.00**	**1.00**	D	**1.00**	**1.00**	D	0.40
2.6.1.1/2.6.1.9	**1.00**	**1.00**	D	**1.00**	**1.00**	D	0.40
2.6.1.62/2.6.1.76	**1.00**	**1.00**	D	**1.00**	**1.00**	D	0.40
2.7.2.11/2.7.2.8	**1.00**	**1.00**	D	**1.00**	**1.00**	D	0.40
2.7.3.2/2.7.3.3	0.96	**1.00**	W	**1.00**	0.96	W	0.70
3.1.1.1/3.1.1.7	0.68	**1.00**	W	**1.00**	0.85	W	0.40
3.5.3.1/3.5.3.8	**0.95**	**0.95**	D	**0.95**	0.94	W	0.40
3.6.3.6/3.6.3.8	**0.98**	0.76	L	0.76	**0.97**	L	0.40
4.1.1.17/4.1.1.20	**1.00**	**1.00**	D	**1.00**	**1.00**	D	0.40
4.2.1.3/4.2.1.33	0.98	**0.99**	W	**0.99**	0.37	W	0.40
4.2.1.3/4.2.1.36	**1.00**	**1.00**	D	**1.00**	0.70	W	0.40
4.3.1.3/4.3.1.5	**1.00**	**1.00**	D	**1.00**	0.93	W	0.40
4.3.2.1/4.3.2.2	**1.00**	0.97	L	0.97	**1.00**	L	0.40
5.1.3.2/5.1.3.20	**1.00**	**1.00**	D	**1.00**	**1.00**	D	0.50
5.4.2.10/5.4.2.2	**0.86**	0.62	L	0.62	**0.96**	L	0.40
6.1.1.11/6.1.1.15	**1.00**	0.97	L	0.97	**1.00**	L	0.50
6.1.1.12/6.1.1.22	0.94	**0.98**	W	**0.98**	0.91	W	0.40
6.1.1.12/6.1.1.6	**1.00**	**1.00**	D	**1.00**	**1.00**	D	0.40
6.1.1.15/6.1.1.3	0.99	**1.00**	W	**1.00**	0.89	W	0.40
6.1.1.17/6.1.1.18	0.98	**1.00**	W	**1.00**	0.98	W	0.60
6.3.2.13/6.3.2.8	**1.00**	**1.00**	D	**1.00**	**1.00**	D	0.40
6.3.2.13/6.3.2.9	0.83	**1.00**	W	**1.00**	0.93	W	0.30
6.3.2.8/6.3.2.9	0.85	**0.98**	W	**0.98**	0.92	W	0.30

F-measures of the nearest neighbour classifier, the ASC classifier and the best classifier based on the full MSA are given in the columns 1NN, ASC and MSA, respectively. The first part of the table compares ASC with the 1NN classifier and the column WDL gives the wins, draws and losses of the pairwise comparisons. Similarly, the second part of the table compares ASC with the SVM classifier based on the full MSA. The last column gives the used MTTSI value.

### Decarboxylating dehydrogenases

To exemplify the interpretability of the ASC models we present a full analysis of the family of decarboxylating dehydrogenases. This family features enzymes that catalyse the dehydration and decarboxylation of several malate derivatives, namely isocitrate, 3-isopropylmalate and tartrate [37]. The substrates have a malate in common but differ in their 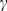-substituents. It was shown that the 3-isopropylmalate dehydrogenase (IPMDH) does not utilise isocitrate as substrate and also that the isocitrate dehydrogenase (ICDH) does not utilise isopropylmalate as substrate [37]. Furthermore, IPMDH shows a relaxed specificity for alkylmalates, and accepts a wide range of substrates alkylated at the 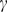-site [37].

We could extract 61, 286 and 8 sequences from SwissProt that were annotated as isocitrate (EC 1.1.1.42), 3-isopropymalate (EC 1.1.1.85) or tartrate dehydrogenases (EC 1.1.1.93), respectively. EC class 1.1.1.83 contains enzymes that are also members of this family and utilise D-malate. But they were not included as a separate class in this set, since these enzymes seem to be equivalent to the enzymes from EC class 1.1.1.93. The tartrate enzymes readily utilise D-malate as substrate, according to the 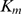 and 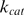 values given by the BRENDA database [38] for the enzymes of class 1.1.1.93. Hence, we put the eight tartrate- and eight D-malate-specific sequences into one class. For the ASC analysis of this set of sequences, we used the crystal structure of a 3-isopropymalate dehydrogenase from *Thiobacillus ferrooxidans* (PDB-Id: 1A05, [37]) as the structural template and extracted all residues within 6 Å of the cognate substrate 3-isopropylmalate. Because it is known that IPMDH forms homo-dimeric complexes, we chose this crystal structure of the homo-dimeric form of the enzyme to enable ASC to select residues from all chains of the enzyme that are in contact with the substrate [37]. In the initial filtering step, 333 sequences could be aligned with sufficient sequence identity to the template sequence and a reliable guide MSA with an overall CORE score of 69 could be built. From this MSA, 17 residues lining the active site were extracted (Table 3). The resulting ASC model, using the string kernel 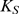, achieved a F-measure of 99% (precision = 99%, recall = 100%) when evaluated at an MTTSI of 60%.

**Table 3:** Decarboxylating dehydrogenases active site signature.

Number	Amino acid	CORE score	Number	Amino acid	CORE score
1	Val73	8	10	Lys190^*^	9
2	Glu88	6	11	Asn192^*^	9
3	Leu91	7	12	Val193^*^	7
4	Leu92	8	13	Asp222^*^	9
5	Arg95	9	14	Asn242	9
6	Arg105	8	15	Asp246	8
7	Arg133	9	16	Asp250	9
8	Leu135	7	17	Glu275	9
9	Tyr140	9			

Residue identifiers are taken from the template crystal structure (PDB-Id: 1A05). Residues highlighted with asterisks are from chain B of the homo-dimeric enzyme. The CORE scores are those from the family MSA generated by 3DCoffee.

### Model interpretation: Isocitrate vs. isopropylmalate specificity

**Figure 2:**
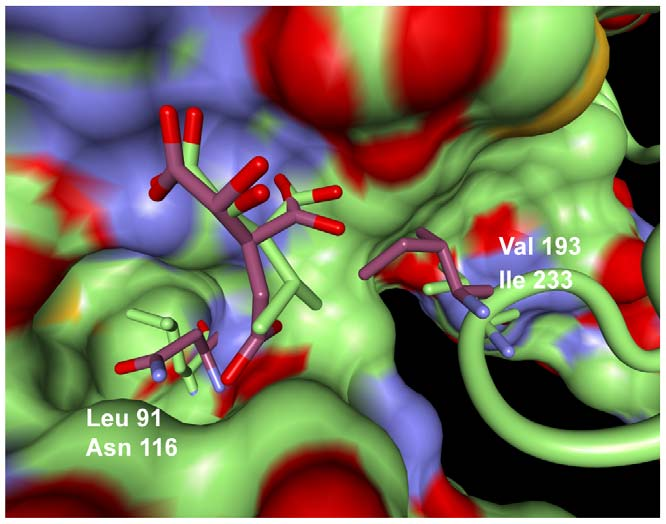
View on the superimposed active sites of IPMDH and ICDH. The first chain of the homo-dimeric enzyme is represented by its solvent-excluded surface. The second chain is depicted in a backbone representation. The two substrates isocitrate (purple) and isopropylmalate (green) lie in the interface of the two chains. IPMDH sidechains are coloured green and sidechains from ICDH (PDB-Id: 1AI2, [40]) are coloured purple. This figure was created using BALLView [41].

The two top-ranked features of the model discriminating isocitrate-specific enzymes from isopropylmalate-specific enzymes were residues Leu91 (accuracy = 98.2%) and Val193 (accuracy = 97.2%) in the template structure 1A05. The enzymes acting on isocitrate, with its charged 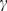-site that accepts hydrogen bonds, prefer asparagine residues at position Leu91. Whereas enzymes acting on isopropylmalate prefer leucine. When inspecting the superimposed structures of the two enzymes shown in Figure 2, the preference of replacing Leu91 with asparagine in the isocitrate dehydrogenase can be explained by the hydrogen bond formed between the carboxylate group at the 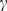-site of isocitrate and the terminal amino group of asparagine. The isopropylmalate enzymes replace the hydrogen bonding asparagine with the hydrophobic Leu91, which can interact optimally with the aliphatic 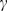-site of isopropylmalate.

At position Val193, the ICDH enzymes prefer an isoleucine whereas IPMDH enzymes prefer valine. One possible explanation for these preferences might be that the isocitrate 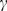-site is more flexible due to the additional carbon atom and is oriented to the asparagine residue at position Leu91. The shorter isopropyl lacks this flexibility and is therefore located near position Val193, where IPMDH enzymes prefer the smaller valine. The larger isoleucine placed by ICDH enzymes might function as a further switch to filter out substrates featuring rigid isopropyl substituents at the 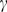-site. The 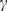-site of the cognate substrate isocitrate of ICDH would orient itself to the asparagine, whereas the isopropyl group of isopropylmalate molecules would clash with the large isoleucine residue that is preferred at position Val193 by the ICDH enzymes.

### Model interpretation: Tartrate vs. isopropylmalate specificity

The two most important residues in the model that discriminates between the substrates tartrate and isopropylmalate were Glu88 (accuracy = 100%) and Val193 (accuracy = 97%). Enzymes specific for tartrate or malate prefer the large amino acid tryptophan at the position corresponding to Glu88, whereas the enzymes specific for substrates with larger substituents at the 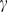-site prefer amino acids like glutamate or arginine. Tryptophan may act as a filter to prevent substrates with larger 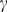-substituents to bind efficiently to the active site. The substitution pattern at site Val193 seems to be coupled to the placement of the tryptophan. Enzymes having a tryptophan at position Glu88 prefer a glycine or alanine at the position corresponding to Val193, probably to prevent steric clashes with the tryptophan now located nearby.

### Availability of the predictive web server

The whole workflow for combining sequence and structural data, building the classification model and interpreting the model parameters in the context of the enzyme's structure is made available to users by the means of a web service. ASC itself is implemented in C++ and makes use of the BALL library to process protein structures [39]. The web server also offers a specialised report page for model interpretation, where the sequence signatures are linked to the structure of the active site in an interactive fashion using a Jmol applet to visualise the structure. A typical result page can be found in the supplementary material (Text S3). When labelled sequence data is scarce a special extract-only mode of ASC can give a first overview of the active site conservation by only extracting the active site signatures. The ASC web service is available at http://asc.informatik.uni-tuebingen.de/.

### Conclusion

We have presented our new method ASC for enzyme sequence classification, which combines sequence data and structural data. By using structural information, we can focus the classification task on features that are most likely relevant for modulating substrate specificity, namely the residues lining the active site. The two benchmarks showed that classification accuracy can be clearly improved by concentrating on the extracted active site signatures and that this improvement is not simply due to the use of SVMs, because the ASC models also outperformed the SVM models trained on the full MSA in many cases. Futhermore, ASC also provides a ranking of the active site residues based on their influence on the decision function. The application of ASC on the benchmark data set by Hannenhalli showed that the set of residues found most relevant for the ASC classification model very often coincided with those residues found relevant by experimental analysis or detected by other computational methods like the sub-profile method. Unlike sequence based classification or FDR-determining methods, ASC had of course the advantage of using structural information and could pre-filter the list of putative specificity modulating residues. Therefore, a direct comparison with those methods would be unfair. However, we think that using this additional source of information is essential for building more accurate function prediction models.

In general, one has to keep in mind that ASC can fail in cases where residues modulate specificity, that are not located in the vicinity of the active site, since the premise of ASC is to use only active site residues. This is not necessarily a limitation of the method though. The absence of any active site signature differences between enzymes with differing class labels can serve as an indication to search for allosteric influences on substrate specificity or to check if the annotation really is correct and both enzymes in reality may be utilising the same substrates or show some cross-specificity.

Applications for our ASC method lie especially in the use of the trained models as predictors of enzymatic function within an enzyme family for sequences that are more remote to the training set and could be predicted more reliably using the extracted active site signatures. But also detection of new subtypes is possible by searching for novel active site signatures in the pool of all extracted signatues of an enzyme family with subsequent experimental validation. Regarding the applicability of ASC, even remote structural homologs might suffice to extract active site residues for novelty detection and classification purposes. Thus, ASC models might be built for many enzyme families to further improve annotation accuracy within current genome annotation pipelines.

Moreover, one can try to verify experimentally the findings gathered in the model interpretation step by mutating residues found relevant to discriminate between two specificities to design mutated enzymes with reduced or even switched substrate specificity. This was exemplified by the dehydrogenase example where we could pinpoint the influence of the residues found relevant on a detailed structural level by inspecting the superimposed active sites of the two enzymes, thereby making predictions of the model more transparent. Finally, our ASC web service allows users to interpret the learned ASC model in the context of the template structure and can yield insight into the mechanisms of substrate specificity by focusing on residues in proximity to the active site and analysing the allowed sequence variation in the corresponding columns of the family MSA.

## Supporting Information

Text S1 ASC performance on Hannenhalli benchmark
(0.08 MB PDF)

Text S2 ASC performance on Capra benchmark
(0.09 MB PDF)

Text S3 ASC web server result page of NRPS dataset
(0.19 MB PDF)
