# Supplementary material for: Combining Structure and Sequence Information Allows Automated Prediction of Substrate Specificities within Enzyme Families
Source: PLoS Comput Biol. 2010 Jan 8;6(1):e1000636. doi: 10.1371/journal.pcbi.1000636 (PMC2796266; doi:10.1371/journal.pcbi.1000636)
Supplement: Text S1 — ASC performance on Hannenhalli benchmark (0.08 MB PDF) [file pcbi.1000636.s001.pdf]

Trypsin				
kernel	F	prec	rec	accu
ASCR	0.64	0.71	0.58	0.78
ASCC	0.90	0.96	0.84	0.91
ASCW	0.90	0.96	0.84	0.91
1NN	0.81	0.84	0.78	0.87
ASCS	0.86	0.92	0.80	0.88
ASCB	0.86	0.92	0.80	0.88
ASCFW	0.80	0.93	0.71	0.83
ASCFS	0.79	0.93	0.69	0.81
ASCFB	0.59	0.59	0.58	0.78
ASCFR	0.59	0.59	0.58	0.78
Dehydrogenase				
kernel	F	prec	rec	accu
ASCR	0.97	0.97	0.96	0.97
ASCC	0.97	0.98	0.97	0.97
ASCW	0.87	0.86	0.89	0.87
1NN	0.94	0.94	0.94	0.95
ASCS	0.96	0.97	0.96	0.97
ASCB	0.98	0.98	0.97	0.98
ASCFW	0.97	0.97	0.97	0.98
ASCFS	0.97	0.97	0.97	0.98
ASCFB	0.98	0.98	0.98	0.98
ASCFR	0.00	0.00	0.00	0.00
Kinase				
kernel	F	prec	rec	accu
ASCR	0.95	0.97	0.94	0.96
ASCC	0.97	0.98	0.96	0.97
ASCW	0.96	0.97	0.94	0.96
1NN	1.00	1.00	1.00	1.00
ASCS	1.00	1.00	1.00	1.00
ASCB	1.00	1.00	1.00	1.00
ASCFW	1.00	1.00	1.00	1.00
ASCFS	1.00	1.00	1.00	1.00
ASCFB	1.00	1.00	1.00	1.00
ASCFR	0.59	0.59	0.58	0.78
Cyclase				
kernel	F	prec	rec	accu
ASCR	0.77	0.82	0.73	0.79
ASCC	0.98	0.98	0.97	0.98
ASCW	0.90	0.89	0.91	0.90
1NN	0.66	0.66	0.66	0.62
ASCS	0.98	0.98	0.97	0.98
ASCB	0.98	0.98	0.97	0.98
ASCFW	0.33	0.29	0.40	0.50
ASCFS	0.31	0.29	0.34	0.41
ASCFB	0.38	0.31	0.49	0.61
ASCFR	0.38	0.31	0.50	0.63
