# Supplementary material for: Combining Structure and Sequence Information Allows Automated Prediction of Substrate Specificities within Enzyme Families
Source: PLoS Comput Biol. 2010 Jan 8;6(1):e1000636. doi: 10.1371/journal.pcbi.1000636 (PMC2796266; doi:10.1371/journal.pcbi.1000636)
Supplement: Text S2 — ASC performance on Capra benchmark (0.09 MB PDF) [file pcbi.1000636.s002.pdf]

		1NN				ASC			ASCF			
EC1	EC2	F	prec	rec	kernel	F	prec	rec	F	prec	rec	mttsi
1.1.1.100	1.1.1.62	0.72	0.82	0.64	B	0.76	0.84	0.69	0.85	0.89	0.81	0.4
1.1.1.103	1.1.1.284	1.00	1.00	1.00	W	1.00	1.00	1.00	1.00	1.00	1.00	0.40
1.1.1.1	1.1.1.103	0.96	0.95	0.97	W	1.00	1.00	1.00	1.00	1.00	1.00	0.40
1.1.1.1	1.1.1.284	0.56	0.56	0.57	B	0.82	0.80	0.83	0.74	0.75	0.74	0.60
1.1.1.41	1.1.1.42	0.94	0.95	0.94	S	0.72	0.79	0.67	0.94	0.95	0.94	0.50
1.1.1.41	1.1.1.85	1.00	1.00	1.00	W	1.00	1.00	1.00	0.96	0.95	0.98	0.60
1.1.1.42	1.1.1.85	0.98	0.97	0.99	W	1.00	1.00	1.00	0.98	0.97	0.99	0.60
1.2.1.3	1.2.1.71	1.00	1.00	1.00	B	1.00	1.00	1.00	1.00	1.00	1.00	0.50
1.2.1.3	1.2.1.8	0.92	0.91	0.92	C	0.93	0.91	0.93	0.83	0.85	0.81	0.60
1.4.1.3	1.4.1.4	1.00	1.00	1.00	W	1.00	1.00	1.00	1.00	1.00	1.00	0.60
1.8.1.4	1.8.1.7	1.00	1.00	1.00	S	1.00	1.00	1.00	1.00	1.00	1.00	0.60
2.1.2.2	2.1.2.9	0.88	0.82	0.94	W	1.00	1.00	1.00	1.00	1.00	1.00	0.30
2.1.3.2	2.1.3.3	0.77	0.67	0.90	W	1.00	1.00	1.00	0.91	0.86	0.98	0.30
2.2.1.1	2.2.1.7	0.99	0.99	0.99	W	1.00	1.00	1.00	0.98	0.98	0.98	0.50
2.3.1.16	2.3.1.9	0.81	0.81	0.82	S	0.93	0.93	0.92	0.96	0.95	0.97	0.50
2.4.2.10	2.4.2.7	0.84	0.80	0.89	S	1.00	1.00	1.00	0.90	0.98	0.83	0.30
2.4.2.22	2.4.2.8	1.00	1.00	1.00	S	1.00	1.00	1.00	1.00	1.00	1.00	0.40
2.4.2.8	2.4.2.9	1.00	1.00	1.00	S	1.00	1.00	1.00	0.91	0.92	0.90	0.40
2.5.1.10	2.5.1.29	0.42	0.41	0.43	W	0.75	0.74	0.77	0.34	0.34	0.34	0.40
2.5.1.1	2.5.1.10	0.52	0.53	0.52	S	0.58	0.59	0.56	0.65	0.70	0.60	0.50
2.5.1.1	2.5.1.29	0.53	0.53	0.61	S	0.77	0.77	0.77	0.73	0.72	0.74	0.50
2.6.1.11	2.6.1.62	1.00	1.00	1.00	S	1.00	1.00	1.00	0.88	0.88	0.88	0.40
2.6.1.11	2.6.1.13	0.94	0.91	0.98	W	0.94	0.91	0.98	0.83	0.88	0.77	0.50
2.6.1.11	2.6.1.76	1.00	1.00	1.00	S	1.00	1.00	1.00	0.46	0.43	0.50	0.40
2.6.1.13	2.6.1.62	1.00	1.00	1.00	S	1.00	1.00	1.00	1.00	1.00	1.00	0.40
2.6.1.13	2.6.1.76	1.00	1.00	1.00	S	1.00	1.00	1.00	1.00	1.00	1.00	0.40
2.6.1.1	2.6.1.9	1.00	1.00	1.00	S	1.00	1.00	1.00	1.00	1.00	1.00	0.40
2.6.1.62	2.6.1.76	1.00	1.00	1.00	S	1.00	1.00	1.00	1.00	1.00	1.00	0.40
2.7.2.11	2.7.2.8	1.00	1.00	1.00	S	1.00	1.00	1.00	1.00	1.00	1.00	0.40
2.7.3.2	2.7.3.3	0.96	0.96	0.97	S	1.00	1.00	1.00	0.96	0.96	0.97	0.70
3.1.1.1	3.1.1.7	0.68	0.70	0.66	S	1.00	1.00	1.00	0.85	0.83	0.88	0.40
3.5.3.1	3.5.3.8	0.95	0.95	0.95	S	0.95	0.95	0.95	0.94	0.94	0.94	0.40

		1NN				ASC			ASCF			
EC1	EC2	F	prec	rec	kernel	F	prec	rec	F	prec	rec	mttsi
3.6.3.6	3.6.3.8	0.98	0.98	0.99	S	0.76	0.81	0.72	0.97	0.96	0.98	0.40
4.1.1.17	4.1.1.20	1.00	1.00	1.00	S	1.00	1.00	1.00	1.00	1.00	1.00	0.40
4.2.1.3	4.2.1.33	0.98	0.98	0.97	S	0.99	0.99	0.99	0.37	0.29	0.50	0.40
4.2.1.3	4.2.1.36	1.00	1.00	1.00	S	1.00	1.00	1.00	0.70	0.57	0.92	0.40
4.3.1.3	4.3.1.5	1.00	1.00	1.00	S	1.00	1.00	1.00	0.93	0.98	0.88	0.40
4.3.2.1	4.3.2.2	1.00	1.00	1.00	S	0.97	0.98	0.96	1.00	1.00	1.00	0.40
5.1.3.2	5.1.3.20	1.00	1.00	1.00	S	1.00	1.00	1.00	1.00	1.00	1.00	0.50
5.4.2.10	5.4.2.2	0.86	0.84	0.87	C	0.62	0.65	0.59	0.96	0.95	0.97	0.40
6.1.1.11	6.1.1.15	1.00	1.00	1.00	W	0.97	0.97	0.97	1.00	1.00	1.00	0.50
6.1.1.12	6.1.1.22	0.94	0.92	0.97	W	0.98	0.97	0.99	0.91	0.93	0.89	0.40
6.1.1.12	6.1.1.6	1.00	1.00	1.00	S	1.00	1.00	1.00	1.00	1.00	1.00	0.40
6.1.1.15	6.1.1.3	0.99	0.99	0.99	W	1.00	1.00	1.00	0.89	0.90	0.88	0.40
6.1.1.17	6.1.1.18	0.98	0.97	0.98	W	1.00	1.00	1.00	0.98	0.97	0.98	0.60
6.3.2.13	6.3.2.8	1.00	1.00	1.00	S	1.00	1.00	1.00	1.00	1.00	1.00	0.40
6.3.2.13	6.3.2.9	0.83	0.83	0.83	S	1.00	1.00	1.00	0.93	0.93	0.93	0.30
6.3.2.8	6.3.2.9	0.85	0.85	0.85	S	0.98	0.98	0.98	0.92	0.92	0.91	0.30
