# Supplementary material for: Combining Structure and Sequence Information Allows Automated Prediction of Substrate Specificities within Enzyme Families
Source: PLoS Comput Biol. 2010 Jan 8;6(1):e1000636. doi: 10.1371/journal.pcbi.1000636 (PMC2796266; doi:10.1371/journal.pcbi.1000636)
Supplement: Text S3 — ASC web server result page of NRPS dataset (0.19 MB PDF) [file pcbi.1000636.s003.pdf]

The Active Site Classification web service

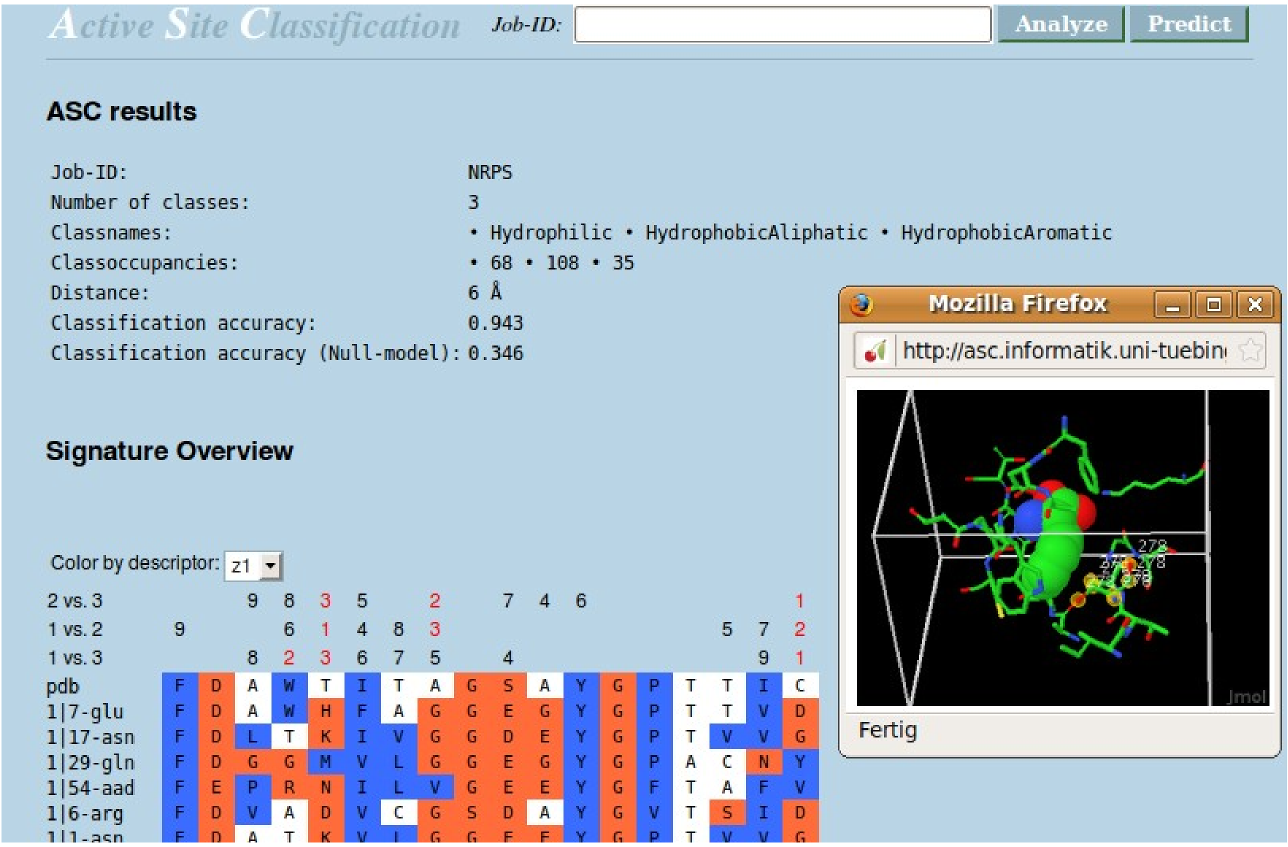

The image shows a screenshot of a typical result page of the ASC web service. Depicted are the validation results from an ASC model trained on the NRPS data set. The problem is a three class problem with NRPS specificities grouped into the three categories hydrophilic (class 1), hydrophobic-aliphatic (class 2) and hydrophobic-aromatic (class 2). Inspection of the residues found most relevant, as given by the rank numbers above each column, for discriminating between class 1 and class 2 (hydrophilic vs. hydrophobic-aliphatic) shows that at signature positions 5 and 18 hydrophilic residues are preferably placed by the class 1 NRPS enzymes to interact optimally with the hydrophilic substrates. Whereas class 2 enzymes preferably place large hydrophobic residues at those positions to optimally interact with any small hydrophobic substrates but also hinder efficient binding of large hydrophobic substrates (namely large aromatic compounds). Similarly, class 3 enzymes preferably place smaller hydrophobic residues at those positions to allow efficient binding of larger aromatic compounds. The popup window on the right hand side contains a Jmol applet that gives an interactive 3D view on the active site of the template structure. When clicking on any signature column within the result page the corresponding residue in the Jmol applet gets highlighted. This feature allows to quickly inspect the important columns and analyse the effect of the placement of certain residues in the structural context of the active site displayed in the Jmol applet.
